# Outcomes of Total Laparoscopic Hysterectomy: A Single-Surgeon Experience at a Tertiary Care Hospital in North India

**DOI:** 10.7759/cureus.79675

**Published:** 2025-02-26

**Authors:** Divya Mishra, Eshna Singh, Shantanu Shubham

**Affiliations:** 1 Obstetrics and Gynaecology, Graphic Era Institute of Medical Sciences, Dehradun, IND; 2 Neonatology, Graphic Era Institute of Medical Sciences, Dehradun, IND

**Keywords:** hysterectomy, minimally invasive laparoscopy, minimally invasive surgery, safe surgery, total laparoscopic hysterectomy

## Abstract

Introduction

Total laparoscopic hysterectomy (TLH) has emerged as a preferred surgical approach for managing various benign and premalignant gynecological conditions. It offers significant advantages over traditional hysterectomy techniques, including reduced blood loss, shorter hospital stays, and lower complication rates. However, its adoption varies across healthcare settings, with challenges such as a steep learning curve and technical difficulties in certain patient populations. This study presents a single surgeon's experience with TLH in a tertiary care center in North India, focusing on perioperative outcomes, complications, and surgical efficiency.

Methods

This retrospective observational study analyzed 150 patients who underwent TLH between January 2022 and December 2024. Data were collected from hospital records, including demographic details, surgical indications, intraoperative findings, perioperative outcomes, and complications. The surgical technique involved a standardized laparoscopic approach, with energy sources like LigaSure™ (Medtronic, Minneapolis, MN, USA) and harmonic probes. Statistical analysis included descriptive measures, with continuous variables presented as means and categorical variables as frequencies.

Results

The mean age of the patients was 50.04 ± 7.42 years, with a mean BMI of 27.77 ± 7.05. The most common indications for TLH were adenomyosis in 39 (26%), leiomyoma in 31 (20.67%), and combined leiomyoma with adenomyosis in 31 (20.67%). The mean operative time was 39 minutes, and the mean blood loss was 25 mL. Perioperative complications were minimal, with only one (0.67%) case each of ureteric stricture, bowel injury, bladder injury, and conversion to laparotomy. ICU admission was required in two (1.33%) cases, while postoperative urinary tract infections occurred in two (1.33%). The mean hospital stay was 2.09 days, and six (4%) patients required readmission.

Conclusion

This study highlights the safety, efficiency, and favorable perioperative outcomes of TLH in a tertiary care setting. The low complication rates, short hospital stays, and minimal blood loss reinforce TLH as a viable alternative to conventional hysterectomy methods. While the study benefits from a large sample size and single-surgeon consistency, its retrospective design and single-center scope are limitations. Future multi-center studies with long-term follow-up are recommended to validate these findings and explore the role of robotic-assisted hysterectomy in improving surgical outcomes.

## Introduction

Total laparoscopic hysterectomy (TLH) has emerged as a preferred surgical approach for the management of various gynecological conditions due to its minimally invasive nature, which offers several advantages over traditional methods such as total abdominal hysterectomy (TAH). In India, while TLH is becoming increasingly common in corporate and private healthcare settings, its adoption in government institutions is still evolving, with surgical audits being infrequent [1].

Globally, TLH is recognized for its benefits, including reduced blood loss, shorter hospital stays, and lower complication rates compared to TAH [2]. Studies conducted in various regions have demonstrated that TLH is a safe and effective procedure, with common indications being abnormal uterine bleeding and leiomyoma [3]. The procedure's success is largely attributed to advancements in surgical techniques and equipment, as well as the increasing experience of surgeons [4,5].

Internationally, TLH has been shown to be feasible across different patient demographics, including those with varying body mass indices and previous surgical histories, such as cesarean sections [6,7]. The learning curve associated with TLH is significant, with studies indicating that surgical outcomes improve as surgeons gain more experience, leading to decreased complication rates and improved patient outcomes [5,8].

Despite being the second most frequently performed procedure in gynecology departments after cesarean section, TLH has not gained widespread adoption. Although it offers significant advantages and favorable clinical outcomes, its prevalence remains limited. In the United States, rather than increasing, the number of TLH procedures has actually declined. Data indicate that TLH accounted for 15.5% of hysterectomies in 2006, but this figure dropped to 8.6% by 2010 [9]. One contributing factor to this trend is the rising preference for robotic-assisted hysterectomy [9]. Additionally, TLH presents certain challenges, such as a steep learning curve for surgeons and technical difficulties, particularly in obese patients [10].

With increasing experience, gynecology consultants have reduced the operative time and complications of TLH. This study presents a single surgeon's experience with 150 TLH cases, analyzing intraoperative and early postoperative outcomes. The results are compared with published literature to reevaluate complications and outcomes. Conducted in a North Indian tertiary care center, this research provides valuable insights into TLH's challenges and benefits, contributing to improved surgical practices and patient safety in diverse healthcare settings.

## Materials and methods

Study design and setting

This was a retrospective observational study conducted at a tertiary care center in North India, analyzing 150 patients who underwent TLH between January 2022 and December 2024. Data were extracted from hospital records, including demographic details, indications for surgery, intraoperative findings, perioperative outcomes, complications, and surgical details.

Inclusion and exclusion criteria

This study included women who underwent TLH for benign or premalignant gynecological conditions, provided they had complete medical records available for review. Patients undergoing TLH for malignant conditions were excluded unless the malignancy was an incidental postoperative histopathological finding. Additionally, cases of hysterectomy performed via abdominal or vaginal approaches were not considered. Patients with incomplete data were also excluded from the study.

Surgical procedure

Following the induction by general anesthesia, the patient was positioned in the low lithotomy position. The bladder was emptied using an infant feeding tube, which was maintained in situ for the entire duration of the surgery. To prevent the leakage of intraperitoneal gas during vaginal vault dissection, a menstrual cup of appropriate size was placed.

**Figure 1:**
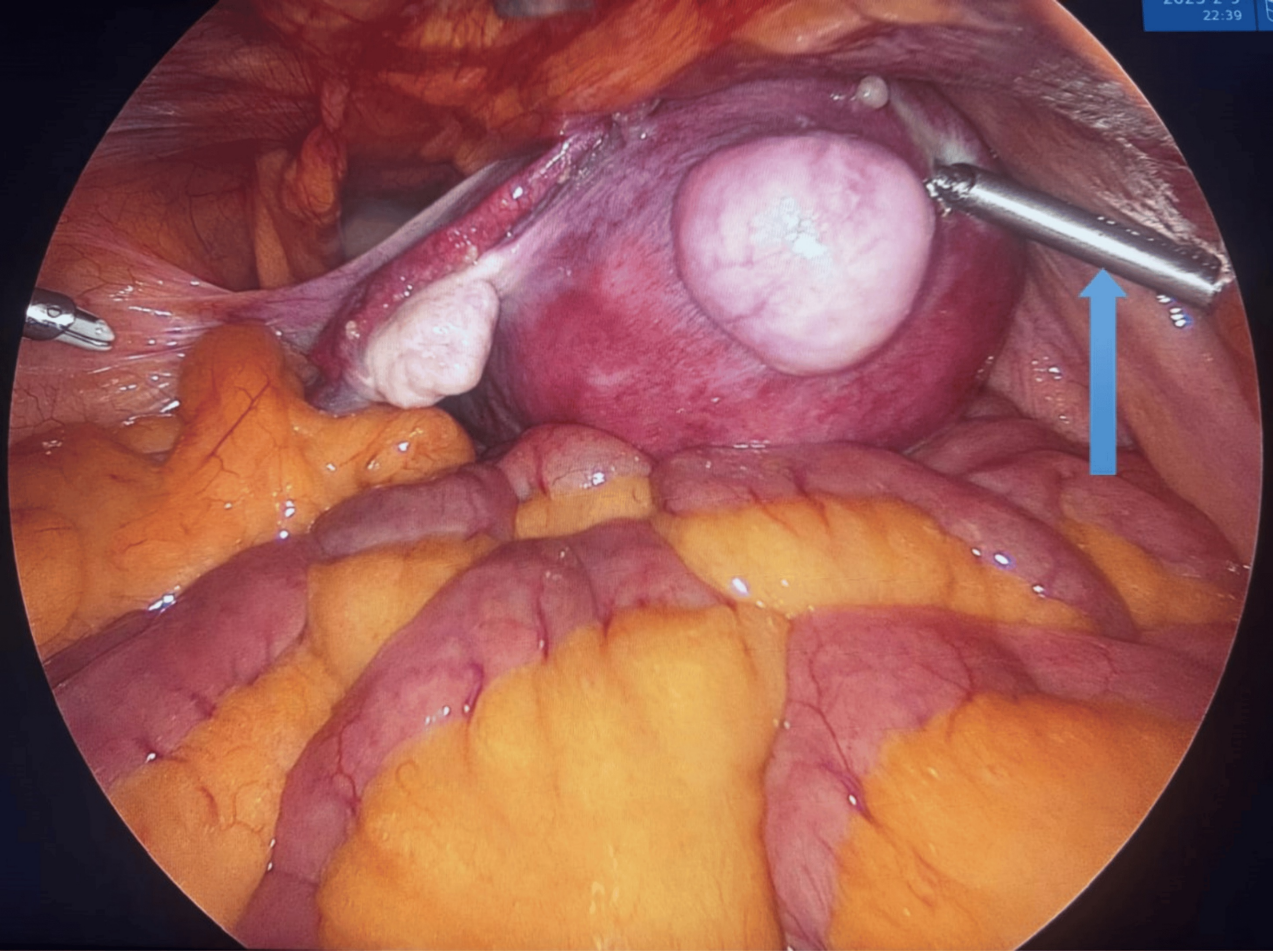
Upward traction to the uterus using a laparoscopic myoma screw (blue arrow)

A 10 mm primary port was inserted using the direct, sharp trocar entry technique at the modified Palmer's point, except in cases with a previous supraumbilical midline vertical scar, where the primary port was placed at Jain's point. Under camera guidance, two 5 mm lateral ports were inserted 2 cm above and medial to the anterior superior iliac spine (ASIS). In cases with an enlarged uterus, these ports were positioned slightly higher. A third 5 mm port was inserted 1-2 cm above the midpoint of the line, joining the two 5 mm lateral ports. A laparoscopic myoma screw was introduced through the right-sided 5 mm port to provide upward traction to the uterus (Figure 1).

The primary energy source used in most cases was LigaSure™ (Medtronic, Minneapolis, MN, USA) with a Maryland tip, while a harmonic probe was utilized in cases of dense endometriotic adhesions. The surgical steps included coagulation and transection of the upper pedicles, followed by bladder dissection, dissection of posterior visceral peritoneum, coagulation and transection of uterine arteries, followed by uterosacral ligaments. Finally, the vaginal vault was opened, and the specimen was delivered vaginally, with manual vaginal morcellation performed if necessary. The closure of the vault was done vaginally.

All the surgeries have been performed by the same surgeon, who has been trained in minimally invasive and pelvic surgeries after a postgraduate degree in Obstetrics and Gynecology. Moreover, to minimize complications and to decrease the time required for surgery, a few steps and techniques have been improvised, e.g., the use of a laparoscopic myoma screw instead of a vaginal manipulator, routine dissection of the posterior peritoneum, Trendelenberg (head-low) position of the patient after creation of the pneumoperitoneum to keep the bowel away from the surgical field, and direct trocar entry technique instead of using a veress needle for the creation of the pneumoperitoneum. In cases of a densely adherent bladder, a retrograde filling of the bladder with normal saline before bladder dissection was performed. The use of Palmer's point for primary trocar entry and Jain's point in cases of midline vertical scars ensured the prevention of trocar-related injuries. These steps were used uniformly in all the surgeries and helped minimize the intraoperative challenges.

Upon completion of the procedure, a Foley catheter was placed after the removal of the feeding tube. Early ambulation was encouraged within four to six hours postoperatively, and a soft diet was initiated after six hours, except in cases requiring bowel adhesiolysis. The bladder catheter was generally removed within 12-24 hours, and patients were typically discharged within 36-48 hours postoperatively.

Data collection and parameters assessed

Patient data were retrospectively retrieved from hospital records and operative notes. The study analyzed demographic and clinical characteristics, including age, parity, body mass index (BMI), anemia status, and the presence of comorbidities such as hypertension, diabetes mellitus, hypothyroidism, chronic kidney disease, hepatic cirrhosis, and hemoglobinopathies. A history of previous lower segment cesarean sections (LSCS) and other laparoscopic and open abdominal surgeries was also recorded. The indications for TLH were categorized into common conditions such as adenomyosis, leiomyoma, endometrial hyperplasia, and prolapse, among others.

Intraoperative details, including operative time (defined as the time from skin incision to the completion of port site closure), estimated blood loss, uterine size, and additional procedures performed (such as salpingectomy, salpingo-oophorectomy, sacropexy, and ureterolysis), were recorded. Perioperative outcomes were assessed by noting intraoperative complications such as bladder, ureter, or bowel injury and the requirement for conversion to laparotomy or ICU admission. Postoperative outcomes, including duration of catheterization, length of hospital stay, incidence of infections, thromboembolic events, and readmission rates, were documented. The study aimed to evaluate both surgical and perioperative outcomes, ensuring a comprehensive assessment of TLH safety and efficacy.

Statistical analysis was performed using STATA, Version 14 (StataCorp LLC, College Station, TX, USA). For statistical analysis, descriptive statistics were employed to summarize findings. Continuous variables were reported as mean ± standard deviation (SD) or median with interquartile range (IQR) where appropriate, while categorical variables were expressed as frequencies and percentages. Ethical approval for the study was obtained from the Institutional Ethics Committee at Graphic Era Institute of Medical Sciences (No.: GEIMS/IRB/RP/26/2025). Patient confidentiality was strictly maintained following institutional and ethical guidelines.

## Results

Demographic and clinical characteristics

A total of 150 patients who underwent TLH were included in the study. The mean age of the patients was 50.04 ± 7.42 years (range: 35-78). The mean parity was 2.53 ± 1.21 (range: 0-6), and the mean BMI was 27.77 ± 7.05 (range: 18.3-48).

Among the patients, 53 (35.33%) had mild anemia, 51 (34%) had moderate anemia, and 24 (16%) had severe anemia. The most common comorbidities other than anemia were hypertension in 64 (42.67%), hypothyroidism in 43 (28.67%), and diabetes mellitus in 24 (16%). Other less frequent conditions included bronchial asthma in six (4%), thalassemia trait in four (2.67%), chronic kidney disease in three (2%), valvular heart disease in two (1.33%), liver cirrhosis in two (1.33%), seizure disorder in one (0.67%), and hepatitis C in one (0.67%). A history of previous LSCS was present in 68 (45.33%) patients, with 41 (27.33%) having undergone one LSCS, 23 (15.33%) two LSCS, and four (2.67%) three LSCS. Additionally, 36 (24%) patients had a history of laparoscopy, and 12 (8%) had undergone laparotomy (Table 1).

**Table 1:** Demographic and clinical characteristics BMI, body mass index; LSCS, lower segment cesarean section

Parameter		Mean (SD)	N (%)	Range
Age (Years)		50.04 (7.42)	-	35–78
Parity		2.53 (1.21)	-	0–6
BMI		27.77 (7.05)	-	18.3–48
Anemia	Mild	-	53 (35.33)	-
	Moderate	-	51 (34)	-
	Severe	-	24 (16)	-
Comorbidities	Hypertension	-	64 (42.67)	-
	Hypothyroidism	-	43 (28.67)	-
	Diabetes Mellitus	-	24 (16)	-
	Bronchial Asthma	-	6 (4)	-
	Thalassemia Trait	-	4 (2.67)	-
	Chronic Kidney Disease	-	3 (2)	-
	Valvular Heart Disease	-	2 (1.33)	-
	Liver Cirrhosis	-	2 (1.33)	-
	Seizure Disorder	-	1 (0.67)	-
	Hepatitis C	-	1 (0.67)	-
Previous LSCS	One	-	41 (27.33)	-
	Two	-	23 (15.33)	-
	Three	-	4 (2.67)	-
Previous Surgeries	Laparoscopy	-	36 (24)	-
	LSCS and Laparoscopy	-	18 (12)	-
	Laparotomy	-	12 (8)	-
	Laparoscopy and Laparotomy	-	9 (6)	-
	LSCS and Laparotomy	-	9 (6)	-

Indications for TLH

The most common indications for TLH were adenomyosis in 39 (26%), leiomyoma in 31 (20.67%), and combined leiomyoma with adenomyosis in 31 (20.67%). Other indications included endometrial hyperplasia in 12 (8%), endometrial polyp in 10 (6.67%), and various degrees of uterovaginal prolapse in 11 (7.33%). Endometriosis in five (3.33%), ovarian cysts in four (2.67%), high-grade squamous intraepithelial lesion (HSIL) in three (2%), endometrial intraepithelial neoplasia in three (2%), and endometritis in two (1.33%) were observed in smaller proportions​ (Table 2).

**Table 2:** Indications for total laparoscopic hysterectomy (TLH) HSIL, high-grade squamous intraepithelial lesion; UV, uterovaginal

Indication	N (%)
Adenomyosis	39 (26)
Leiomyoma	31 (20.67)
Leiomyoma + adenomyosis	31 (20.67)
Endometrial hyperplasia	12 (8)
Endometrial polyp	10 (6.67)
Second-degree UV prolapse	6 (4)
First-degree UV prolapse	5 (3.33)
Endometriosis	5 (3.33)
Ovarian cyst	4 (2.67)
HSIL	3 (2)
Endometrial intraepithelial neoplasia	3 (2)
Endometritis	2 (1.33)

Perioperative outcomes and complications

Among the 150 TLH procedures, there were no cases of ureteric injury, vesicovaginal fistula, ureterovaginal fistula, peritonitis, paralytic ileus, deep vein thrombosis, or pulmonary embolism. One case (0.67%) each of ureteric stricture, bowel injury, bladder injury, and conversion to laparotomy was noted. ICU admission was required in two (1.33%), and one (0.67%) required blood transfusion postoperatively. Postoperative urinary tract infection (UTI) was observed in two (1.33%), while no cases of vaginal cuff infection or trocar site infection were reported. The mean postoperative catheter duration was 1.19 days (range: 1-14 days), and the mean hospital stay was 2.09 days (range: two to seven days). There was one case (0.67%) of histopathologically diagnosed malignancy postoperatively, and six (4%) required readmission (Table 3)​. Despite the observed variations, the overall complication rates in this study were low. Consequently, no statistically significant differences were found in complication rates between patients with high BMI and those with normal BMI, nor between patients with prior surgical history and those without. The lack of significance suggests that the study may be underpowered to detect meaningful differences, highlighting the need for a larger sample size to draw more definitive conclusions.

**Table 3:** Perioperative outcomes and complications ICU, intensive care unit; UTI, urinary tract infection

Complications	N (%)
Ureteric stricture	1 (0.67)
Bowel injury	1 (0.67)
Bladder injury	1 (0.67)
Conversion to laparotomy	1 (0.67)
ICU requirement	2 (1.33)
Blood transfusion	1 (0.67)
Post-op UTI	2 (1.33)
Histopathology diagnosis of malignancy	1 (0.67)
Post-op readmission	6 (4)
-	Mean (Range)
Post-op catheter duration (days)	1.19 (1–14)
Post-op hospital stay (days)	2.09 (2–7)

Surgical outcomes and operative details

The majority of cases involved TLH with bilateral salpingectomy in 68 (45.33%) and TLH with bilateral salpingo-oophorectomy in 68 (45.33%). TLH with right or left salpingo-oophorectomy was performed in seven (4.67%) cases each. Ureterolysis was required in three (2%) cases, and sacropexy was performed in five (3.33%) cases. Only one (0.67%) required re-operation, and no mortality was observed. The mean uterine size was 11.8 cm (range: 6-28 cm), the mean operative time was 39 minutes (range: 27-130 minutes), and the mean blood loss was 25 mL (range: 15-320 mL)​ (Table 4). Power morcellation was not done in any surgery. The longer duration of the surgery in a few cases can be attributed to either manual vaginal morcellation of a large-size uterus or encountering dense endometriotic adhesions in some cases requiring ureterolysis and bowel adhesiolysis.

**Table 4:** Surgical outcomes and operative details TLH, total laparoscopic hysterectomy; B/L, bilateral; BSO, bilateral salpingo-oophorectomy; RSO, right salpingo-oophorectomy; LSO, left salpingo-oophorectomy

Parameter		N (%)
Type of surgery	TLH + B/L salpingectomy	68 (45.33)
	TLH + BSO	68 (45.33)
	TLH + RSO	7 (4.67)
	TLH + LSO	7 (4.67)
Ureterolysis		3 (2)
Sacropexy		5 (3.33)
Need for re-operation		1 (0.67)
-		Mean (Range)
Uterine size (cm)		11.8 (6–28)
Operating time (minutes)		39 (27–130)
Blood loss (mL)		25 (15–320)

## Discussion

TLH has gained prominence as a minimally invasive alternative to TAH, offering superior perioperative outcomes, including reduced blood loss, shorter operative time, and lower postoperative morbidity.

The perioperative outcomes of TLH have been extensively studied, with numerous reports indicating improved patient safety and reduced intraoperative complications. In our study, the mean estimated blood loss was 25 mL (range: 15-320 mL), aligning with findings from Puntambekar et al. (2020), who reported a slightly higher blood loss in 1200 TLH cases [4]. Similarly, Tantia et al. (2024) documented a mean blood loss of nearly 30 mL in a study involving 773 TLH cases, reinforcing the hemostatic benefits of advanced laparoscopic techniques [5]. Another study documented the average blood loss to be 140 mL. Around 10% of patients had an estimated blood loss of less than 10 mL, as there was barely any visible bleeding during the procedure, minimal to no oozing from the laparoscopic incision sites, and almost no blood pooling in the pelvic cul-de-sac at the end of the surgery. Furthermore, more than half of the patients had a total blood loss of under 50 mL [1]. A retrospective study of 361 cases by Mereu et al. (2018) documented a mean blood loss of 44 mL during TLH for benign disease [11].

The mean operating time for TLH has been reported to vary significantly across different studies, ranging from 86 to 150 minutes [12-16]. In contrast, the mean operating time in our study was significantly lower, at 39 minutes. Several studies have demonstrated improved surgical efficiency with increasing experience and training. The use of advanced energy sources for coagulating and transecting tissues also aids significantly in reducing the total operating time and blood loss during surgery. McDonnell et al. (2018) conducted a retrospective study analyzing over 2000 TLH cases at a single institution and reported a mean operating time of 61 minutes [10]. Their findings also highlighted that operative time improved progressively with the surgeon's learning curve and advanced training in gynecologic surgery. Similarly, Bhandari et al. (2014) investigated the impact of BMI on TLH and reported a mean operating time of 54 minutes [17]. Another study by Zakaria et al. (2023) compared TLH with abdominal hysterectomy for benign gynecological conditions and found the mean operating time for TLH to be 55 minutes [18].

In our study, the most common indications for TLH were adenomyosis in 39 (26%), leiomyoma in 31 (20.67%), and a combination of leiomyoma with adenomyosis in 31 (20.67%). Other indications included endometrial hyperplasia in 12 (8%), endometrial polyp in 10 (6.67%), uterovaginal prolapse in 11 (7.33%), and endometriosis in five (3.33%). These findings align with previous studies that have identified abnormal uterine bleeding, leiomyoma, and adenomyosis as the primary indications for TLH [4,5]. Studies have also highlighted the increasing utilization of TLH for women with previous cesarean sections, demonstrating its feasibility and safety in this population [6,7].

Our study reported a mean postoperative hospital stay of 2.09 days (range: two to seven days), which is consistent with findings from other studies indicating an average stay of 1.5 to three days for TLH [10,17]. The short hospital stay observed in our study may be attributed to early mobilization, standardized postoperative pain management protocols, and minimally invasive techniques, which reduce surgical trauma and facilitate faster recovery. Studies have demonstrated that laparoscopic hysterectomy is associated with a significantly shorter hospital stay compared to abdominal hysterectomy, further reinforcing its benefits [16,18].

The complications encountered in this study were notably low, with no recorded cases of vesicovaginal fistula, ureterovaginal fistula, peritonitis, paralytic ileus, deep vein thrombosis (DVT), or pulmonary embolism. However, isolated intraoperative and postoperative complications were observed. Ureteric stricture was noted in one (0.67%) case, which is consistent with the reported incidence of ureteral injuries in TLH ranging from 0.2% to 1.5% in previous studies [1,2]. Bladder injury occurred in one (0.67%) case, a rate comparable to prior literature findings that estimate the incidence between 0.2% and 2%, particularly in patients with prior pelvic surgeries such as cesarean sections [4]. Similarly, bowel injury, a rare but serious complication, was documented in one (0.67%) case, aligning with reported rates of 0.2% to 1% in TLH procedures [10,12]. Conversion to laparotomy was required in one (0.67%) case, which is lower than the previously reported 1.5% to 4% conversion rates observed in large-scale reviews [11].

Postoperatively, two (1.33%) required ICU admission, likely due to perioperative complications or underlying comorbidities, which aligns with expected rates seen in previous studies [5]. Postoperative blood transfusion was required in only one (0.67%) case, further supporting the hemostatic benefits of laparoscopic hysterectomy, with prior research indicating transfusion rates between 0.5% and 3% [14]. Additionally, postoperative UTI was observed in two (1.33%) patients, which is comparable to the 1% to 2% incidence reported in TLH literature [13]. Readmission was necessary for six (4%) cases, primarily due to minor postoperative complications, which is consistent with previously documented TLH readmission rates ranging from 3% to 6% [16].

This study on TLH has several strengths, including a large sample size of 150 cases, which provides a strong dataset for analysis. The single-surgeon experience ensures consistency in surgical techniques, minimizing variability in outcomes. Additionally, the comprehensive perioperative assessment offers valuable insights into both intraoperative and postoperative outcomes, while the study's low complication rates reinforce the safety and efficacy of TLH in a tertiary care setting. Furthermore, the comparison with existing literature enhances its relevance.

However, there are limitations, including its single-center, single-surgeon design, which restricts generalizability to other institutions and surgeons. The retrospective nature of the study makes it susceptible to selection and information biases, and the lack of long-term follow-up limits understanding of outcomes beyond the immediate postoperative period. This study focuses on perioperative and immediate postoperative outcomes without long-term follow-up. However, complications such as vaginal cuff dehiscence, chronic pelvic pain, or adhesions may develop later and could influence the overall assessment of TLH. The absence of long-term data limits our ability to fully evaluate the procedure's long-term safety and effectiveness.

## Conclusions

In conclusion, this study highlights the safety, efficacy, and favorable perioperative outcomes of TLH in a tertiary care setting. The low complication rates, short hospital stays, and minimal blood loss reinforce TLH as a viable alternative to conventional hysterectomy methods. While the study benefits from a large sample size and single-surgeon consistency, its retrospective and single-center designs are limitations. Future research with multicenter data and long-term follow-up is needed to further validate these findings and explore advancements such as robotic-assisted hysterectomy to enhance surgical outcomes and patient care.
